# Association between diabetic retinopathy in type 2 diabetes and the *ICAM-1* rs5498 polymorphism: a meta-analysis of case-control studies

**DOI:** 10.1186/s12886-018-0961-5

**Published:** 2018-11-12

**Authors:** Zikang Xie, Hao Liang

**Affiliations:** Department of Ophthalmology, First Affiliated Hospital, Guangxi Medical University, Nanning, China

**Keywords:** ICAM-1, rs5498, Meta-analysis, Diabetic retinopathy

## Abstract

**Background:**

Genetic studies have reported contradictory results on the association between the intercellular adhesion molecule-1 (*ICAM-1*) rs5498 polymorphism and diabetic retinopathy (DR) risk in type 2 diabetic patients. We aimed to perform a systematic literature search and conduct random-effects meta-analysis to provide a quantitative evaluation.

**Methods:**

We searched Pubmed, Embase, Scopus, Web of Science and Wanfang databases from inception up to January 2018. Allelic and genotype frequencies of rs5498 was compared between DR cases and controls. Odds ratios (OR) and 95% confidence intervals (CI) were calculated using a random effects model.

**Results:**

Nine studies involving a total of 1792 cases and 1400 controls met our inclusion criteria. We did not find any significant association between rs5498 and DR risk at the dominant model (GG + GA versus AA, OR = 1.00, 95% CI: 0.66–1.50, *P* = 0.987), the recessive model (GG versus GA + AA, OR = 1.24, 95% CI: 0.86–1.77, *P* = 0.245), the GG versus AA contrast (OR = 1.14, 95% CI: 0.68–1.92, *P* = 0.611), and the G allele versus A allele contrast (OR = 1.08, 95% CI: 0.81–1.45, *P* = 0.592). Subgroup analysis by ethnicity showed no association in Asian populations (G allele versus A allele: OR = 1.05, 95% CI: 0.76–1.44, *P* = 0.790). Subgroup analysis by DR subtype also did not reveal any association of rs5498 with proliferative DR (G allele versus A allele: OR = 1.34, 95% CI: 0.71–2.52, *P* = 0.364) and non-proliferative DR (G allele versus A allele: OR = 0.71, 95% CI: 0.43–1.17, *P* = 0.180).

**Conclusion:**

Our meta-analyses provide no evidence of the association of rs5498 with DR in type 2 diabetic patients.

**Electronic supplementary material:**

The online version of this article (10.1186/s12886-018-0961-5) contains supplementary material, which is available to authorized users.

## Background

Diabetic retinopathy (DR) is the single most common complication of diabetes mellitus and the leading cause of blindness in working-aged adults worldwide [1]. Despite numerous breakthroughs in the development of novel pharmacological agents for DR in the last decade, the incidence of DR remains high and 90% of type 1 and 60% of type 2 diabetes patients suffer from the disease. Body mass index (BMI), increased duration of diabetes, ineffective blood glucose control, and ineffective blood pressure control are the major risk factors for DR [1, 2]. However, they do not adequately predict disease progression in individual patients, suggesting the presence of a genetic component. Identification of the specific genetic risk factors for DR susceptibility is an area of substantial research and could unravel druggable targets for the purpose of treatment or even prevention.

In recent years, emerging evidence has highlighted the potential role of intercellular adhesion molecule-1 (ICAM-1) in the development of DR. ICAM-1 is a immunoglobulin-(Ig)-like transmembrane glycoprotein expressed on the surface of leukocytes, endothelial cells, and epithelial cells [3]. It influences the adhesion of circulating immune cells to the endothelium and contributes to immune cell migration and perivascular infiltration. Increased levels of ICAM-1 and its ligands have been observed in patients with DR and retina of animal models [4–7]. ICAM-1 blockade with monoclonal antibodies effectively prevents diabetic retinal leukostasis, vascular leakage, and capillary nonperfusion in experimental DR [4]. Similarly, when the bioactivity of the ICAM-1 counter receptor CD18 is inhibited, diabetic retinal leukocyte adhesion is potently suppressed [5].

Because ICAM-1 has been implicated in DR development, multiple studies have investigated how genetic variation at *ICAM-1* is related to DR risk. More than 100 single-nucleotide polymorphisms (SNPs) were identified in the *ICAM-1* gene. The best studied SNP is a G/A polymorphism in exon6 at codon 469 (rs5498), resulting in a lysine (Lys) to glutamine (Glu) substitution in Ig-like domain 5 that is essential for dimerisation, surface presentation and solubilisation of the protein [8]. This polymorphism has been shown to influence the interaction of ICAM-1 with leukocyte function-associated antigen-1 (LFA-1) and the macrophage-1 antigen during leukocyte adhesion [8]. In the present study, we aimed to provide a quantitative evaluation of the association between DR in type 2 diabetes and the *ICAM-1* rs5498 polymorphism.

## Methods

### Literature search

The search strategy for this meta-analysis was comprehensive, aiming to retrieve the largest possible number of relevant studies. We systematically screened 5 electronic databases including Pubmed (Additional file 1), Embase, Scopus, Web of Science and Wanfang for articles published between January 1990 and January 2018. The following keywords were used: intercellular adhesion molecule-1, K469E, rs5498, diabetic retinopathy, type 2 diabetes, and polymorphism. In addition, the reference lists of all the retrieved papers and relevant reviews were manually searched for eligible papers. We only included published studies with full-text articles available. In case of overlap between articles reporting on the same cohort, we included the study with the largest cohort. Our meta-analysis adhered to the Preferred Reporting Items for Systematic Reviews and Meta-Analyses (PRISMA) Statement (Additional file 2) [9].

### Inclusion and exclusion criteria

One reviewer performed the initial screen of all papers identified by the electronic searches. Studies were excluded when the title clearly indicated that it did not meet the inclusion criteria. Where a title/abstract could not be rejected with certainty, the full text of the publications was obtained for assessment. Studies were considered eligible if they met the following criteria: 1) evaluated the frequency of the *ICAM-1* rs5498 polymorphism in relation to the number of retinopathy cases and controls; 2) published in English or Chinese; and 3) published prior to January 2018 unless an online version of the study had been released prior to this date; and 4) reported odds ratios (ORs) and 95% confidence intervals (95% CIs) or data to calculate them. Case-only and case series studies with no control population were excluded, as well as studies based only on phenotypic tests, reviews, meta-analysis. We also excluded unpublished studies or gray literature because we expected them to contain insufficient reporting for our analysis.

### Data extraction and quality assessment

Data extraction was performed by the first author and entered into predesigned electronic tables. The second author checked the extracted data. Disagreements were resolved by discussion between the two authors. The following items were considered: first author, year of publication, location of the study, ethnicity, number of cases and controls, diagnostic criteria, allele or genotype frequency, Hardy-Weinberg equilibrium (HWE) status, and genotyping method. The methodological quality of each study was assessed by the Newcastle-Ottawa Scale, which was used for its simplicity in comparing observational studies. Studies were evaluated based on cohort selection, comparability and ascertainment of exposure using nine multiple-choice questions. Studies were deemed of low quality if the total score was 5 or lower [10].

#### Statistical analyses

Statistical analysis was performed using STATA 11 (StataCorp, College Station, TX). To assess HWE status, we used a publicly available program (http://ihg.gsf.de/cgi-bin/hw/hwa1.pl). For our main analysis, we compared allele frequencies (the -encoding allele G versus the -encoding allele A) between cases and controls. We also evaluated a dominant model (GG + AG versus AA) and a recessive model (GG versus AG + AA) for the G allele. All associations were presented as ORs with their corresponding 95% CIs. Heterogeneity was evaluated by Cochran’s Q and the I^2^ statistic. When heterogeneity was high (I^2^ > 50%, *P* < 0.10), a pooled analysis was conducted using DerSimonian and Laird random effects models [11]. The significance of the summary OR was determined using an asymptotic Z-test. We evaluated publication bias or selective reporting by using funnel plots as well as Egger’s regression intercept test.

## Results

### Study characteristics

**Fig. 1:**
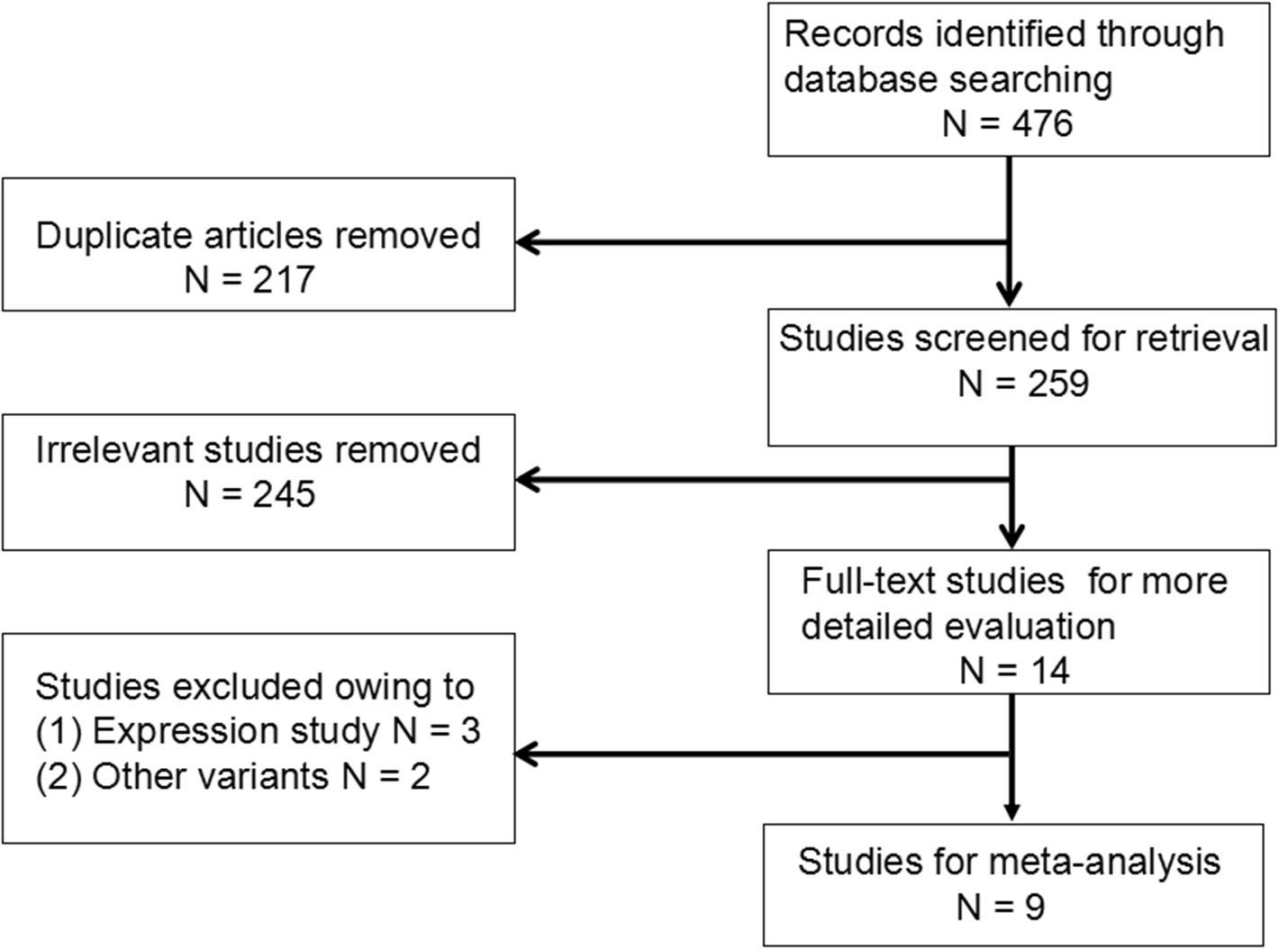
Flow diagram of studies considered for inclusion

Our search yielded 476 records, with 14 articles being possibly eligible after review on abstract level. After full-text review, we excluded 5 studies. Overall, 9 studies involving 1792 cases and 1400 controls met the selection criteria and could be used for meta-analysis [12–20]. Figure 1 showed the process of identifying eligible studies. The mean (range) year of publication was 2010 (2002–2016). The mean (range) sample size was 355 (70–792). Studies had been performed in 4 countries, including China (*n* = 5), India (*n* = 2), Japan (*n* = 1), and Slovenia (*n* = 1). Study characteristics and methodological quality of included studies are shown in Table 1.

**Table 1:** Characteristics of the included studies

First author	Year	Country	Ethnicity	Female percentage (%)		HWE	Age		NOS	Method of DR ascertainment	Genotyping method	Cases (n)	Controls (n)	MAF (%)
				Cases	Controls		Cases	Controls						
Kamiuchi	2002	Japan	Asians	52.0	56.8	Yes	64.3 ± 8.9	64.1 ± 9.1	7	Ophthalmoscopy and fluorescein angiography	PCR-RFLP	81	50	50.0
Liu	2006	China	Asians	PDR: 66.3 NPDR: 67.3	50.0	Yes	PDR: 55.0 ± 12.4 NPDR: 63.7 ± 7.9	50.2 ± 10.6	7	NA	DNA sequencing	132	80	38.8
Petrovic	2008	Slovenia	Caucasians	53.3	60.1	Yes	65.2 ± 9.9	66.9 ± 11.5	7	Fundus photographs	Allele-specific PCR	195	143	42.3
Zhou	2010	China	Asians	49.0	48.0	No	55.6 ± 8.8	55.3 ± 8.6	6	Ophthalmoscopy and fluorescein angiography	PCR-RFLP	102	150	33.0
Zhu	2010	China	Asians	NA	NA	Yes	NA	NA	6	Ophthalmoscopy and fluorescein angiography	PCR-RFLP	40	30	16.7
Balasubbu	2010	India	Asians	30.0	42.0	Yes	57 ± 9	59 ± 11	7	Ophthalmoscopy and fundus photographs	SNaPshot PCR	345	359	48.2
Vinita	2012	India	Asians	35.7	37.6	Yes	58.8 ± 8.6	64.3 ± 9.0	8	Fundus photographs	DNA sequencing	199	157	54.8
Lv	2016	China	Asians	56.3	52.6	Yes	62.4 ± 11.9	60.2 ± 11.7	7	Fundus photographs	PCR-LDR	448	344	29.4
Li	2016	China	Asians	PDR: 51.7 NPDR: 46.2	43.8	Yes	PDR: 66.9 ± 6.9 NPDR: 63.1 ± 6.8	54–82	7	NA	DNA sequencing	250	87	41.4

*DR* diabetic retinopathy, *HWE* Hardy-Weinberg equilibrium, *MAF* minor allele frequency, *NA* not available, *NOS* Newcastle-Ottawa scale, *NPDR* non-proliferative diabetic retinopathy, *PCR-LDR* polymerase chain reaction-ligase detection reaction, *PCR-RFLP* polymerase chain reaction-restriction fragment length polymorphism, *PDR* proliferative diabetic retinopathy

#### Data synthesis

**Fig. 2:**
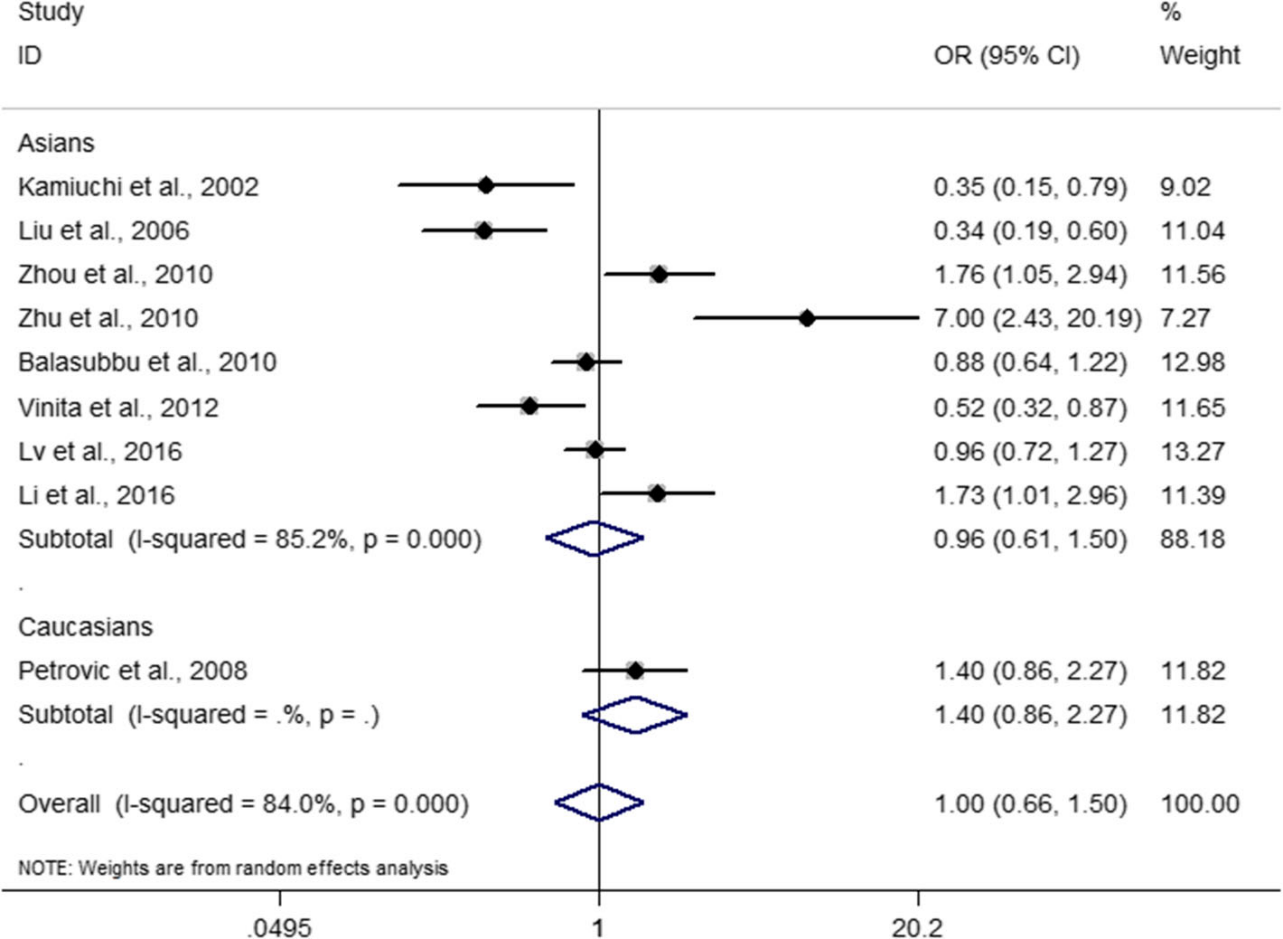
Forest plot for included studies evaluating the association between the *ICAM-1* rs5498 polymorphism and diabetic retinopathy in type 2 diabetic patients under a dominant model (GG + AG versus AA). OR, oadds ratio; CI, confidence interval

**Fig. 3:**
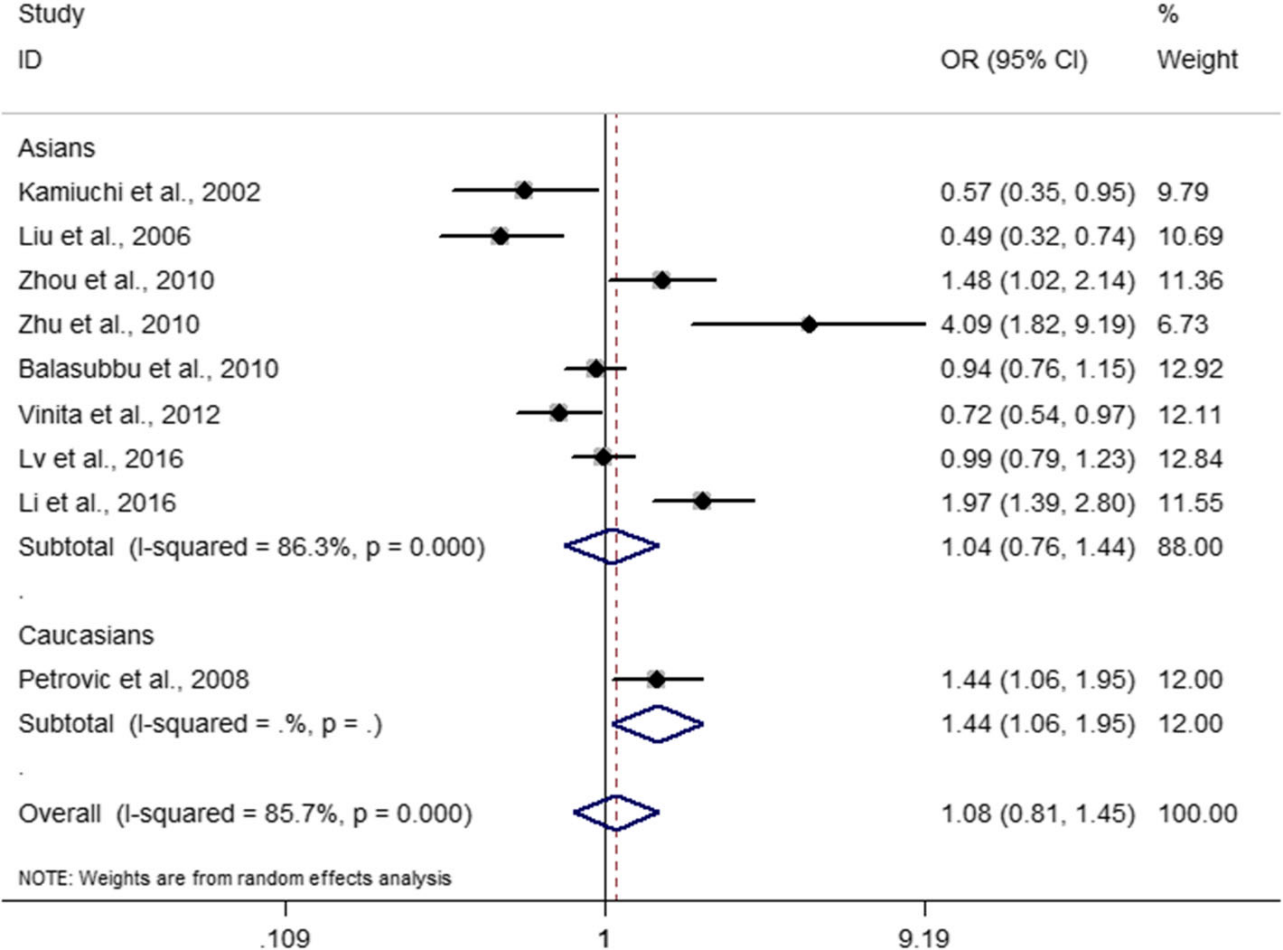
Forest plot for included studies evaluating the association between the *ICAM-1* rs5498 polymorphism and diabetic retinopathy in type 2 diabetic patients under allele contrast (G allele versus A allele). OR, oadds ratio; CI, confidence interval

**Fig. 4:**
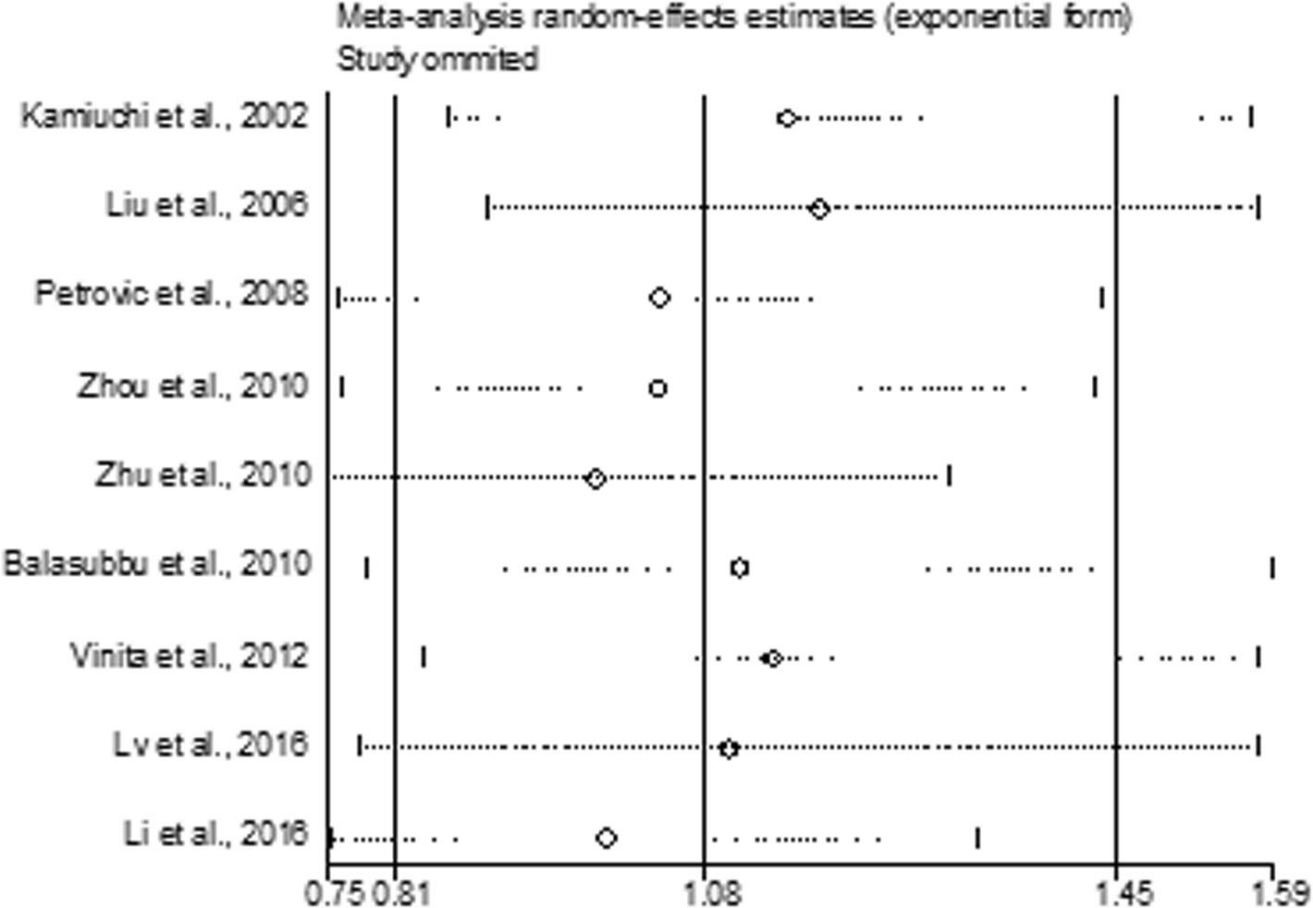
Influential analysis for the *ICAM-1* rs5498 polymorphism to examine the robustness of the overall estimation. ICAM-1, intercellular adhesion molecule-1

The minor allele frequency (MAF) for the *ICAM-1* rs5498 polymorphism varied from 16.7 to 54.8%. The pooled effect estimates among all studies did not find statistically significant associations between the *ICAM-1* rs5498 polymorphism and retinopathy in type 2 diabetes at the dominant model (GG + GA versus AA, OR = 1.00, 95% CI: 0.66–1.50, *P* = 0.987), the recessive model (GG versus GA + AA, OR = 1.24, 95% CI: 0.86–1.77, *P* = 0.245), the GG versus AA contrast (OR = 1.14, 95% CI: 0.68–1.92, *P* = 0.611), and the G allele versus A allele contrast (OR = 1.08, 95% CI: 0.81–1.45, *P* = 0.592) (Table 2 and Figs. 2 and 3). Among the included studies, 8 studies with 1597 cases and 1257 controls were performed on Asian populations. We conducted subgroup analyses by ethnicity using Asian studies, but we did not find any significant associations of the *ICAM-1* rs5498 polymorphism with retinopathy in Asians (dominant model: OR = 0.96, 95% CI: 0.61–1.50, *P* = 0.843; recessive model: OR = 1.15, 95% CI: 0.79–1.68, *P* = 0.469; GG versus AA contrast: OR = 1.04, 95% CI: 0.60–1.81, *P* = 0.887; G allele versus A allele: OR = 1.05, 95% CI: 0.76–1.44, *P* = 0.790) (Table 2 and Figs. 2 and 3). The single Caucasian study showed a statistically significant association between rs5498 and retinopathy at the recessive model (OR = 2.00, 95% CI: 1.15–3.48, *P* = 0.014), the GG versus AA contrast (OR = 2.21, 95% CI: 1.16–4.22, *P* = 0.016) and the G allele versus A allele contrast (OR = 1.44, 95% CI: 1.06–1.95, *P* = 0.021) (Table 2) [14]. When DR was subdivided into non-proliferative DR and proliferative DR, subgroup analysis did not show evidence of significant associations (Table 2). The influential analysis for the *ICAM-1* rs5498 polymorphism revealed that there was no single study which significantly influenced the overall results (Fig. 4).

**Table 2:** Meta-analysis of the association between rs5498 and DR in type 2 diabetes

Evaluation	Number of studies	OR (95% CI)	P	P for heterogeneity	I^2^ (%)
GA + GG versus AA					
Total	9	1.00 (0.66–1.50)	0.987	< 0.001	84.0
Asians	8	0.96 (0.61–1.50)	0.843	< 0.001	85.2
Caucasians	1	1.40 (0.86–2.27)	0.173	NA	NA
PDR	4	1.22 (0.61–2.47)	0.577	< 0.001	86.8
NPDR	3	0.60 (0.29–1.21)	0.151	0.007	80.0
GG versus GA + AA					
Total	9	1.24 (0.86–1.77)	0.245	0.003	65.3
Asians	8	1.15 (0.79–1.68)	0.469	0.008	63.2
Caucasians	1	2.00 (1.15–3.48)	0.014	NA	NA
PDR	4	1.90 (0.80–4.50)	0.146	< 0.001	84.2
NPDR	3	0.92 (0.58–1.47)	0.724	0.477	0.0
GG versus AA					
Total	9	1.14 (0.68–1.92)	0.611	< 0.001	78.5
Asians	8	1.04 (0.60–1.81)	0.887	< 0.001	78.1
Caucasians	1	2.21 (1.16–4.22)	0.016	NA	NA
PDR	4	1.91 (0.64–5.73)	0.246	< 0.001	87.6
NPDR	3	0.77 (0.47–1.25)	0.286	0.171	43.5
G allele versus A allele					
Total	9	1.08 (0.81–1.45)	0.592	< 0.001	85.7
Asians	8	1.05 (0.76–1.44)	0.790	< 0.001	86.3
Caucasians	1	1.44 (1.06–1.95)	0.021	NA	NA
PDR	4	1.34 (0.71–2.52)	0.364	< 0.001	82.5
NPDR	3	0.71 (0.43–1.17)	0.180	0.011	77.9

*CI* confidence interval, *DR* diabetic retinopathy, *NA* not applicable, *NPDR* non-proliferative diabetic retinopathy, *OR* odds ratio, *PDR* proliferative diabetic retinopathy

#### Heterogeneity and publication bias

**Fig. 5:**
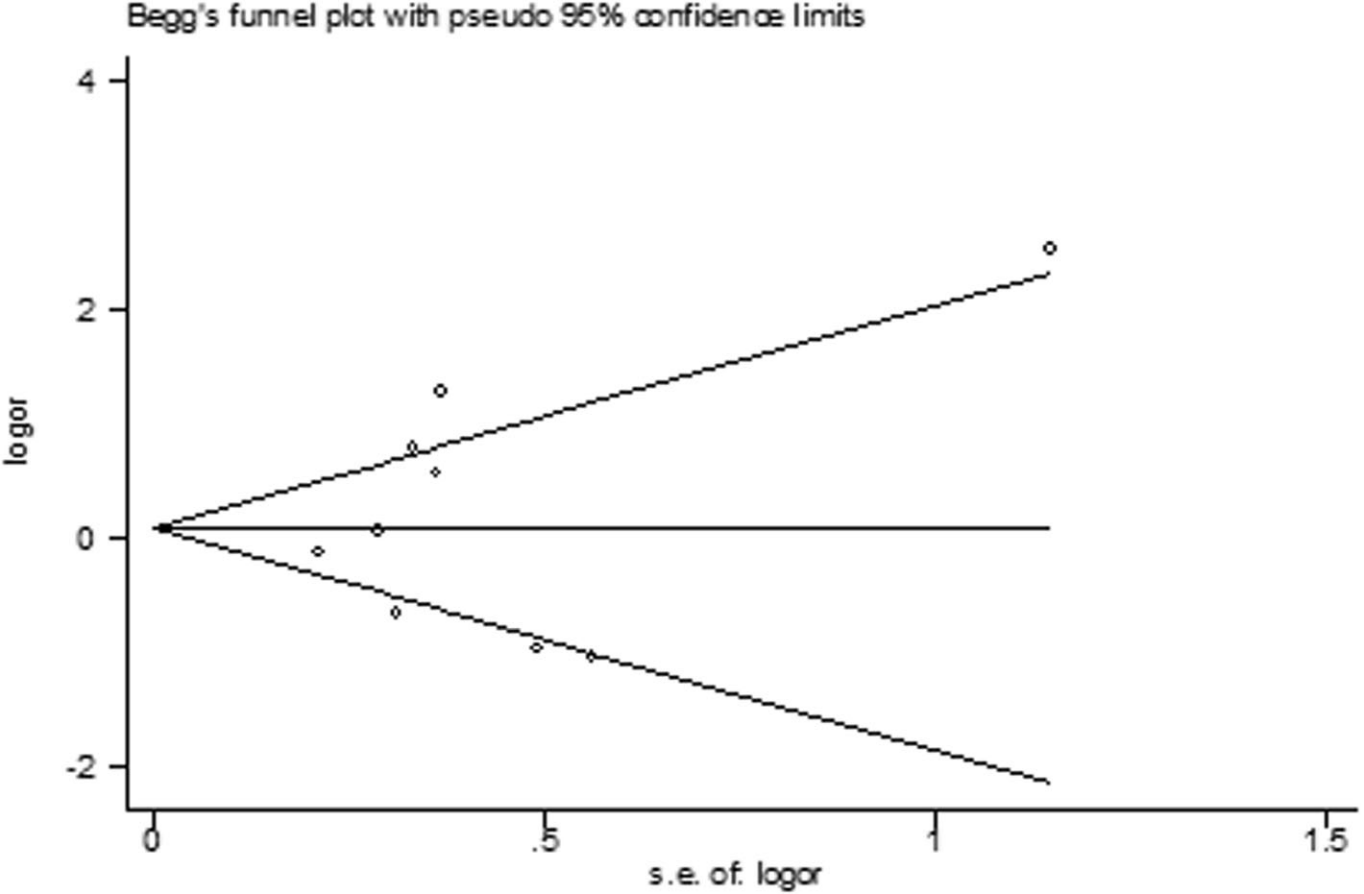
Funnel plot analysis for evaluating publication bias of the association between rs5498 and diabetic retinopathy in type 2 diabetic patients

The present meta-analysis revealed heterogeneity among the included studies (I^2^ ranged from 65.3–84.0%). The funnel plot did not demonstrate apparent asymmetry (Fig. 5). Egger’s test also did not indicate any evidence of publication bias (Table 3).

**Table 3:** Assessment of publication bias using Egger’s test

	Dominant model	Recessive model	GG versus AA contrast	G allele versus A allele contrast
*P* value	0.754	0.498	0.641	0.579

## Discussion

DR is the most frequent microvascular complication from type 2 diabetes. A large body of clinical and experimental literature has indicates that leucocyte adhesion to the retinal vasculature plays an important role in the pathogenesis of DR. As a central mediator of leukocyte adhesion to and transmigration across the endothelium, the gene encoding ICAM-1 is thought to be involved in the development of DR.

The current mete-analytic review was conducted to verify the genetic contribution of a common SNP, rs5498 in the *ICAM-1* gene to retinopathy risk in type 2 diabetes. Our results showed a lack of association between the *ICAM-1* rs5498 polymorphism and risk of retinopathy in type 2 diabetes. Subgroup analysis by ethnicity did not reveal any significant association in Asian populations. In addition, when DR were subdivided into two main stages: non-proliferative and proliferative, we found no association of the *ICAM-1* rs5498 polymorphism with the risk of non-proliferative and proliferative DR.

The *ICAM-1* rs5498 polymorphism results in substitution of an A with a G nucleotide and replaces lysine (K) with a glutamic acid (E). It is thought that the SNP affects mRNA splicing patterns that modify cell-cell interactions and influence inflammatory response [8]. Kamiuchi et al. initially reported a positive association between rs5498 genotypes and retinopathy in type 2 diabetes, using a very small sample size (81 cases and 50 controls) [12]. However, their findings were not replicated by all other studies on the topic. It was noteworthy that the study by Balasubbu et al. with 704 participants and the study by Lv et al. involving 782 participants were the two having relatively large sample sizes among the included studies, but they did not identify any statistically significant association of rs5498 with DR [17, 19]. We could not exclude the possibility that false-positive findings may be obtained from studies with small sample sizes like one conducted by Kamiuchi et al.

Combining published data from nine studies involving 3192 participants, this is the largest meta-analysis on the relationship between the *ICAM-1* rs5498 polymorphism and retinopathy in type 2 diabetes. Previous meta-analyses on the same topic included fewer studies (Su et al., 2013, *n* = 5; Sun et al., 2014, *n* = 7; Fan et al., 2015, *n* = 7) [21–23]. Results from this meta-analysis differed from those of the meta-analysis by Su et al., which found an association between rs5498 and DR in type 2 diabetes. This was probably because in addition to the five studies included by Su et al. [21], we included four recently published case-control studies and conducted the pooled analyses with a larger sample size [22, 23]. Sun et al. and Fan et al. found no association of rs5498 with DR; their results were consistent with our calculations. Compared to the previous meta-analyses, our study had several strengths. First, considering that the cause and development of type 1 diabetes and type 2 diabetes were different, we only included retinopathy subjects of type 2 diabetes as cases in our analyses. We did not take into account the results from type 1 diabetes. Second, in addition to subgroup analyses by ethnicity, we performed subtype-specific analyses to evaluate the relation of rs5498 with the risk of non-proliferative and proliferative DR, respectively. Such evaluations were not performed by the previous meta-analyses. Third, we performed influential analysis to ensure the robustness of our combined estimations.

Since most of the included studies were conducted on Asian populations (*n* = 8), it became evident from this meta-analysis that further studies should include larger non-Asian populations to evaluate race-specific effects of rs5498, such as African and Caucasian populations. The study by Petrovic et al. was the only one performed in Caucasians [14]; their findings should be replicated in other Caucasian populations, including British, German, and French populations. Besides rs5498, more than 200 polymorphisms have been identified in the *ICAM-1* gene. We could not exclude the possibility that other *ICAM-1* polymorphisms played a role in the susceptibility to type 2 DR. The study by Simões et al. identified a significant association between the *ICAM-1* rs1801714 polymorphsim and non-proliferative DR in type 2 diabetes [24]. In addition, a Chinese study assessed the association of DR with the rs1799969 polymorphism which was located in exon 4 of the *ICAM-1* gene [25]. Because the evidence base for other *ICAM-1*polymorphisms was very small at the time of planning our meta-analysis and consequently we chose to focus our analysis only on the rs5498 polymorphism. It would be advisable that in the future attention should be paid to the relationship between other *ICAM-1* polymorphisms and DR in type 2 diabetes.

Several limitations should be considered. First, this study was limited by the unavailability of individual patient data that would allow the identification of potential interactions of rs5498 with specific disease characteristics, including glycemic control, blood pressure control, and hyperlipemia. These factors might have contributed to the lack of success in identifying positive results in association studies for rs5498. Second, like all meta-analyses, the present study was susceptible to reporting biases. Despite the use of comprehensive strategies to identify eligible studies for rs5498, we could not exclude the possibility that some studies might have been erroneously excluded. Third, we did not evaluate the relation of serum soluble ICAM-1 (sICAM-1) levels with DR because of limited published data. There was some evidence suggesting that circulating sICAM levels were positively associated with DR prevalence in type 2 diabetes [14, 26]. It is recommended that future association studies should perform sICAM-1 measurement and evaluate the effects of rs5498 on sICAM levels in DR patients, which could help clarify the role of rs5498 in DR development.

In conclusion, Our meta-analysis has shown that there is no significant association of the *ICAM-1* rs5498 polymorphism with DR in type 2 diabetes. Further investigation involving non-Asian populations is warranted on the association between this polymorphism and DR, particularly studies with larger sample size that adjust for confounding variables.

## Additional files

Additional file 1: Search strategy used in Pubmed. (DOC 27 kb)

Additional file 2: PRISMA Checklist. (DOC 57 kb)

## Abbreviations

BMI: Body mass index
CI: Confidence interval
DR: Diabetic retinopathy
HWE: Hardy-Weinberg equilibrium
ICAM-1: Intercellular adhesion molecule-1
MAF: Minor allele frequency
OR: Odds ratio
SNP: Single-nucleotide polymorphism
