# Supplementary material for: Association between diabetic retinopathy in type 2 diabetes and the ICAM-1 rs5498 polymorphism: a meta-analysis of case-control studies
Source: BMC Ophthalmol. 2018 Nov 12;18:297. doi: 10.1186/s12886-018-0961-5 (PMC6233373; doi:10.1186/s12886-018-0961-5)
Supplement: Supplementary file 1 — Search strategy used in Pubmed. (DOC 27 kb) [file 12886_2018_961_MOESM1_ESM.doc]

Table S1 Search strategy used in Pubmed

| Database |  |
| --- | --- |
| Pubmed | (“mellitus diabetes” [Title/Abstract] OR “mellitus diabetes” [MeSH Terms] OR “diabetes mellitus, type 2” [Title/Abstract] OR “diabetes mellitus, type 2” [MeSH Terms] OR “intercellular adhesion molecule-1”[Title/Abstract] OR “intercellular adhesion molecule-1” [MeSH Terms] OR “polymorphism” [Title/Abstract] OR “polymorphism, genetic” [MeSH Terms] OR “diabetic retinopathy” [Title/Abstract] OR “diabetic retinopathy” [Title/Abstract] ) |
